# Measuring Health Vulnerability: An Interdisciplinary Indicator Applied to Mainland Portugal

**DOI:** 10.3390/ijerph16214121

**Published:** 2019-10-25

**Authors:** Gisela M. Oliveira, Diogo Guedes Vidal, Maria Pia Ferraz, José Manuel Cabeda, Manuela Pontes, Rui Leandro Maia, José Manuel Calheiros, Esmeralda Barreira

**Affiliations:** 1 UFP Energy, Environment and Health Research Unit (FP-ENAS), University Fernando Pessoa, 4249-004 Porto, Portugal; gisela@ufp.edu.pt (G.M.O.); diogovidal@ufp.edu.pt (D.G.V.); mpferraz@ufp.edu.pt (M.P.F.); jcabeda@ufp.edu.pt (J.M.C.); mpontes@ufp.edu.pt (M.P.); rlmaia@ufp.edu.pt (R.L.M.); jcalheiros@ufp.edu.pt (J.M.C.); 2 Health Sciences Faculty, University Fernando Pessoa, 4200-150 Porto, Portugal; 3 Lung Clinic—Portuguese Oncology Institute Francisco Gentil, EPE (IPO-Porto), 4200-072 Porto, Portugal

**Keywords:** health inequalities, health outcomes, health determinants, sustainable development goals, composite indicators, rural–urban disparity, spatially distribution

## Abstract

Health promotion and inequality reduction are specific goals of the United Nations 2030 Agenda, which are interconnected with several dimensions of life. This work proposes a composite index *SEHVI*—socioeconomic health vulnerability index—to address Portuguese population socioeconomic determinants that affect health outcomes. Variables composing *SEHVI* are aligned with the sustainable development goals considering data and times series availability to enable progress monitoring, and variables adequacy to translate populations’ life conditions affecting health outcomes. Data for 35 variables and three periods were collected from official national databases. All variables are part of one of the groups: Health determinants (social, economic, cultural, and environmental factors) and health outcomes (mortality indicators). Variables were standardized and normalized by “Distance to a reference” method and then aggregated into the *SEHVI* formula. Several statistical procedures for validation of *SEHVI* revealed the internal consistency of the index. For all municipalities, *SEHVI* was calculated and cartographically represented. Results were analyzed by statistical tests and compared for three years and territory typologies. *SEHVI* differences were found as a function of population density, suggesting inequalities of communities’ life conditions and in vulnerability to health.

## 1. Introduction

Equity in health was defined by Margaret Whitehead in 1991, and is an utmost issue related to an unfair and uneven distribution of healthcare [1]. In the scope of the United Nations 2030 Agenda for sustainable development goals (SDGs), health promotion—SDG 3—is a multidimensional target directly related to other goals, such as inequality reduction—SDG 10—which emphasizes its complexity [2,3]. It is widely recognized that health outcomes have a strong association with socioeconomic position, becoming more evident in unequal societies and in dense urban areas [4,5,6,7]. Individual health condition is no longer considered only as a consequence of biological causes as previous studies [5,8,9,10,11] indicate that it is also influenced by social, environmental, cultural, political, and economic factors. These factors include housing quality, access to healthcare and education, work conditions, environmental quality, sanitation availability, and safety [12,13].

Welfare states, namely in Europe, play an important role in the development of policies and strategies aiming to reduce health vulnerabilities and inequalities through social transfers and provision of services [7,14,15]. The biggest public health policy challenge is to promote universal health services, which results in healthier communities. This promotion should be supported through education and health literacy that ensure healthy lifestyles [16,17,18,19]. European Health 2020 policy focuses on the reduction of health gaps that are directly associated with social determinants [20]. The objective is to reduce health differences and disparities within and across groups [21,22]. The worst and most visible outcome of health disparities is death by avoidable causes, resulting from absent, delayed, or inadequate healthcare services [10,23,24,25].

The concept of “fundamental causes” of death was developed stating that socioeconomic status is the main cause of inequality in mortality [26,27]. Within this concept, socioeconomic status embodies an availability of resources, such as money, knowledge, prestige, power, and beneficial social connections that may provide relevant mechanisms, at any given time, that facilitate access to specialized medical services. The World Health Organization analyzed health determinants at a multiscale level, deriving the following key findings [28]:

▪ Social factors determine populations health;
▪ Social determinants of health produce inequalities;
▪ Disadvantaged groups are more vulnerable to disease, die younger and experience worse health conditions;
▪ Plans and actions to fight against health inequalities imply a multidisciplinary approach due to its complexity;
▪ Development of instruments to measure social determinants in health are useful to provide knowledge to design tailored public health policies;
▪ Monitoring is very important; thus, health inequalities diagnosis should not be the goal but the starting point of health plans and policies;
▪ Health policies’ effects should be understood as a way to evaluate effectiveness in reducing inequalities.

The Portuguese National Health Service (NHS) was founded with the purpose to assure universal access to healthcare, independently of one’s socio and economic condition, including foreigners, stateless, and political refugees [29]. Despite the gains of the NHS public health policies intervention in Portugal, recent studies [30,31,32] reveal a mismatch between services offered and populations needs. This gap is aggravated in rural and isolated areas where aged, poorly educated, and unemployed groups are predominant. 

This paper addresses the vulnerability in health aligned within the scope of the UN Sustainable Development Goals: “no one is left behind”. For decades, investments in healthcare infrastructures and professionals of NHS have been focused on densely populated urban centers to cover growing population needs, neglecting isolated and rural areas. However, the systematic phenomenon of depopulation, observed for decades, is worsening in rural areas. Response to acute medical emergencies is rather difficult in areas far from densely urban centers where specialized healthcare services are located, therefore specifically affecting the underprivileged communities of rural areas. Recent international studies have highlighted that the human right to health must consider, among other factors, that the universal health “coverage should be on the basis of need, with extra weight given to the needs of the underprivileged” [33,34].

Further research on national health vulnerabilities and recommendations for policies to cope with this issue remains a challenge due to the scarcity of data. Furthermore, the difficulty of accessing periodic data needed to design longitudinal studies is another limitation. Alongside these difficulties, Portugal health’s inequalities are dispersed across the country, justifying a local-scale approach to study this subject.

In this context, the present work proposes a composite index—socioeconomic health vulnerability index (*SEHVI*)—built with several indicators related to health determinants and health outcomes. *SEHVI* was applied on three different years over a nine-year period to assess the evolution of the Portuguese reality: 2009 is the onset of a financial and economic crisis; in 2015, the United Nations declared the 2030 Agenda of Sustainable Development; and 2017/2018 correspond to the last data available on the national databases.

## 2. Materials and Methods 

### 2.1. Study Area

This study has a target area of 89,102 km² that includes the municipalities (N = 278) of mainland Portugal, a European southeastern country, located in the Iberian Peninsula. The Portuguese archipelagos of “Madeira” and “Açores” were excluded from the present study due to the scarcity of available statistical data for the selected variables.

According to National Statistics Institute [35], three territory typologies were defined: Predominantly urban territories (n = 33) are characterized by population density higher than 500 inhabitants/km^2^; semi-urban territories (n = 76) correspond to regions of 100 to 500 inhabitants/km^2^; and in predominantly rural territories (n = 169), population density is under 100 inhabitants/km^2^. 

### 2.2. Model Construction and Data Collection

*SEHVI* was designed in the scope of the United Nations 2030 Agenda for Sustainable Development with the purpose to evaluate how socioeconomic and environmental determinants constrain life conditions, namely health and wellbeing outcomes. Variables selection was supported by several official documents [36,37,38]. This selection took into consideration the availability of data, time-series to enable monitoring progress in time, and the adequacy of the variables in order to aggregate measurable indicators that translate populations’ life conditions. The main goal of *SEHVI* is to analyze the Portuguese reality at local level (municipalities), based on demographic, education, income, housing, work, environmental conditions, and culture investments. Local specificities and constraints are different across the country, making the diagnosis of local life conditions a much-needed goal. Data were collected from different Portuguese official statistic databases—INE (National Statistics Institute), PORDATA (Contemporary Portuguese database), and APA (Environment Portuguese Agency)—and disaggregated at the municipal level. A database was created to analyze gaps in time series and missing values for all variables. At the municipal scale, only 35 variables combined availability of data for the entire mainland geographical coverage and for a timeline.

A total of 35 individual variables were selected and organized in two components as presented in Table 1: Health outcomes, which are related to mortality indicators; and health determinants, relating to social, economic, cultural, and environmental factors to which populations are exposed and that, consequently, condition their health status. A positive (+) or negative (−) character was attributed to each variable in accordance with the impact of that variable in population life conditions and in sustainable natural resources use. As an example: The variable “Selective Urban Waste collection” was classified as positive because waste is duly separated by material type and then directed to reuse, to recycling facilities or to energy valorization. On the contrary, the variable “Undifferentiated Urban Waste collection” contributes negatively to the environment, because urban waste is collected in the same container, without any material type differentiation, and then disposed in a landfill. Variables concerning consumption, such as water supplied/consumed, have a negative polarity because within the sustainable concept of development, consumption has to be penalized [39]. Many other desirable variables, considered relevant to address life conditions, would also have been included if data were available at the local scale. Among others, other variables related to health dimensions [16] would refer to: (i) Built environment—availability and use of cultural, recreational, sports, and green infrastructures; (ii) lifestyles—physical activity, leisure occupation, and nutritional intake; (iii) dependencies—alcohol, smoking, and chemical. All these dimensions are recognized [40] to affect health conditions such as obesity, high blood pressure, and metabolic disorders. 

Furthermore, concerning “health outcomes” dimension, morbidity variables would have been considered if data were available. Data on morbidity of noncommunicable diseases such as obesity or high blood pressure are of extreme importance and strongly related to lifestyles and life conditions. However, Portuguese databases do not provide this kind of information disaggregated at the local level adopted in this study. Table 1 presents all *SEHVI* variables.

### 2.3. SEHVI Formula

The methodology adopted to design the composite index *SEHVI* was the same as previously used to construct another index already published: The *WeGIx* [40,41]. The procedures to standardize, normalize, and aggregate the variables in a non-weighted aggregation formula for the composite index and the statistical approach to validate both indexes were the same. Nevertheless, *SEVHI* and *WeGIx* indexes studies’ objectives, their correspondent variables, period of observation, and geographical coverage are very different. To enable aggregation, a standardization process was applied to each variable presented in Table 1: By resident population in each municipality or by the geographical area of the correspondent municipality. Equations (1) and (2) translate these standardization steps.

Standardization of variables by the resident population in a municipality *i*:

(1) $$I_{i,j}=\frac{v_{i,j}}{P_{i}};\;i\in \left[1,\;278\right];\;j\in \left[1,\;35\right]$$

where:

$I_{i,j\;}$ is the standard value of a variable *v_j_* for a municipality *i*;
$P_{i}$ is the resident population (number of inhabitants) in a municipality *i*;
$v_{i,j}$ is the value of a variable *v_j_* for a municipality *i*.

Standardization of variables by the geographical area of a municipality *i*:

(2) $$I_{i,j}=\;\frac{v_{i,j}}{A_{i}};\;i\in \left[1,\;278\right];\;j\in \left[1,\;35\right]$$

where:

$I_{i,j}$ is the standard value of a variable *v_j_* for a municipality *i*;
$A_{i}$ is the geographical area (km^2^) of a municipality *i*;
$v_{i,j}$ is the value of a variable *v_j_* for a municipality *i*.

Some variables’ raw data were already standardized in their database formats, as is the case of variables expressed as “rate” such as “Infant mortality rate”. For these situations, no standardization procedure was applied. The normalization method adopted for *SEHVI* (Equation (3)) is of the type “Distance to a reference” [42]. For each year of analysis, the reference value for every standardized variable $I_{i,j}$ is the correspondent national mean value $AV_{jP}$.

Normalization by “Distance to a reference” method was used for all indicators [42]:

(3) $$NI_{i,j}=\;\frac{I_{i,j}}{AV_{j,P}};\;i\in \left[1,\;278\right];\;j\in \left[1,\;35\right]$$

where:

$I_{i,j\;}$ is the value of the standard individual indicator *I_j_* for the municipality *i* in a certain year;
$AV_{jP\;}$ is the value of the indicator *I_j_* for Portugal (mean value for the country) for a certain year;
$NI_{i,j}\;$ is the normalized value of the indicator *I_j_* for the municipality *i*.

For each of the 35 individual indicators $NI_{Portugal,j}=1$. For every municipality *i*, each $NI_{i,j}$ indicator is a relative value to the national reference that is always one for each year of analysis. Interaction between all individual indicators $NI_{i,j}$ was statistically tested and analyzed in order to build up a coherent composite model integrating just the necessary dimensions [43,44,45]. The 35 indicators ($NI_{i,j}$) were combined by a simple linear additive method of aggregation using the addition of the arithmetic means (13 positive $NI_{p|i}{}^{+}$ and 22 negative $NI_{n|i}{}^{-}$ indicators), according to Equation (4) [46,47]:

(4) $$SEHVI_{|i}=\frac{\sum_{p=1}^{13}NI_{p|i}{}^{+}}{13}-\frac{\sum_{n=1}^{22}NI_{n|i}{}^{-}}{22};\;i\in \left[1,\;278\right].$$

For each year of analysis, *SEHVI* value for Portugal always has a reference value of zero $SEHVI_{|Portugal}=0\;$ and the $SEHVI_{|i}$ value for each municipality *i* floats above or below the reference. For a specific municipality *i*, if $SEHVI_{|i}>0$, then the correspondent population experiences better health status than the national average. On the other hand, if $SEHVI_{|i}<0$, it means that the correspondent population health is more vulnerable than the national average.

### 2.4. Statistical Analysis

All data calculation and statistical analyses were performed using Excel and IBM^®^ SPSS^®^ Statistics vs. 25.0 software. All cartographic representation of results was performed with ArcGIS™ (Esri).

Variance analysis by *ANOVA* was applied to health determinants and health outcomes indicators and to *SEHVI* in order to compare the results obtained in three territory typologies and for the three time periods. The Tukey test was chosen to compare indicators means at a 0.05 significant statistical level. Scatterplots with health determinants and outcomes by territory typology were elaborated to enable scores comparison of municipalities by urban, semi-urban, and rural typologies. Variances of health determinants and health outcomes were calculated to evaluate results dispersion.

A factor analysis of exploratory type was performed for the internal validation *SEHVI* aiming to explain the variance of the results. Several tests were used to assess the suitability of the respondent data for factor analysis [40,41]. The Kaiser–Meyer–Olkin (KMO) sampling adequacy test was used to measure data quality and the Bartlett’s test of sphericity was used to verify relations between variables and whether the matrix of correlations in the population is an identity matrix. Cronbach’s Coefficient alpha (α) test was applied for reliability analysis. Spearman’s rank correlation coefficient was used to identify associations among variables as well as to assess the convergent validity of *SEHVI* index, using *WeGIx* index to correlate.

## 3. Results and Discussion

### 3.1. SEHVI Validation

Several statistical tests were applied to assess the index construction methodology [40,41,43,45,48]. Results of the factor analysis presented in Table 2 reveals the adequacy of the indicators set through Kaiser–Meyer–Olkin measure of sampling adequacy (KMO = 0.77 > 0.5) and Bartlett’s test (χ^2^ = 7064.2; *p* < 0.001). The factor loads are above 0.30, ranging between 0.31 and 0.93, indicating a high level of validity of the selected items (variables included in *SEHVI*). From the set of 35 indicators integrating the index, nine components were extracted, altogether accounting for 68.2% of the total variance. All communality values are above 0.40, highlighting a great variability proportion for each variable, which is explained by the extracted factors. Regarding the assessment of the *SEHVI* internal consistency, Table 2 shows a value of 0.81 for the global Cronbach’s alpha (α), which is considered high [49]. If any indicators were to be removed from the model, in some cases, it would not change the internal consistency of the index i.e., “8 Number of healthcare professionals”; however, in several cases, the index would become weaker (which, for example, is the case of atmospheric emissions PM, NO_X_, CO_2_).

The convergent validity test shows that *SEHVI* is highly correlated with *WeGIx* (r_s_ = 0.98–0.99, *p* < 0.001), enlightening a strong convergence of the two indexes, an expected result. As *WeGIx* was designed to measure the populations’ global wellbeing at the local level, it was thus expected that municipalities where *WeGIx* scores are higher are also those where *SEHVI* scores are higher too, corresponding to a lower health vulnerability. These results reaffirm SEHVI as a reliable tool for the purpose it was designed.

### 3.2. SEHVI Application

**Figure 1:**
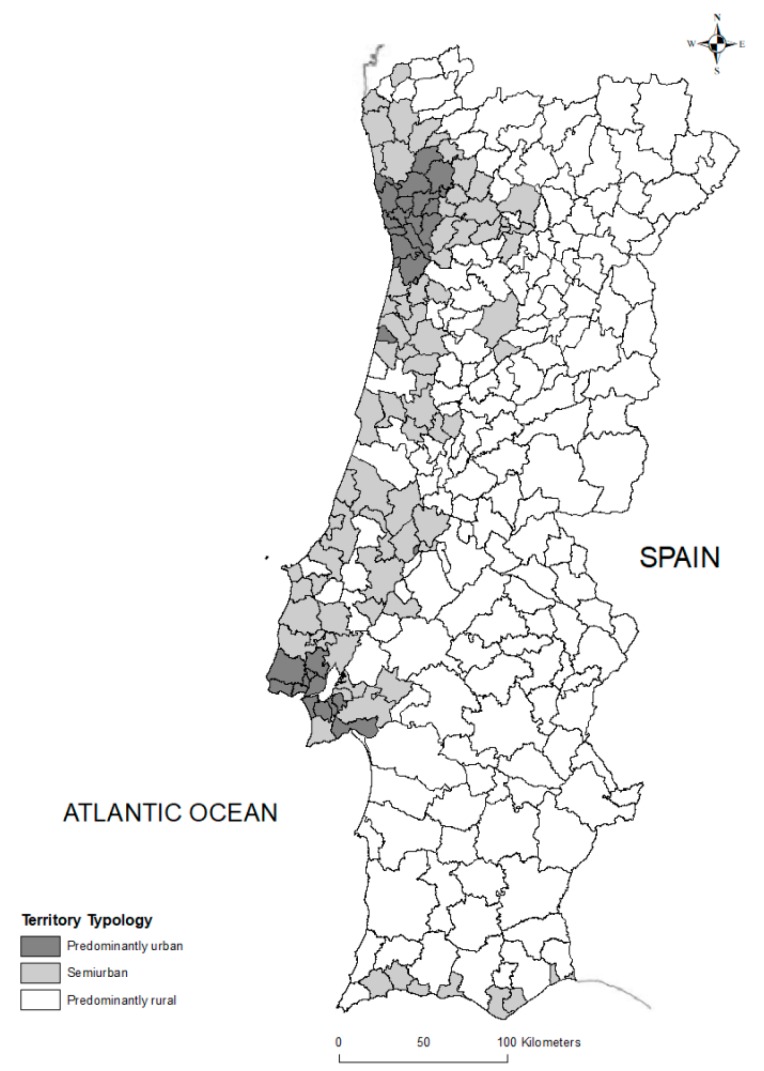
Classification of Portuguese municipalities by population density according to National Statistics Institute (INE) [35].

The cartographic representation of municipalities by population density is presented in Figure 1.

The country is predominantly rural corresponding to 78.5% of the mainland area, with a low population density (29.4 inhabitants/km^2^). Figure 1 also highlights the country demographic imbalances: A littoralization (greyscale), marked by the concentration of population along the coast from the north to the center of mainland; and a bipolarization (dark greyscale), visible by the population concentration in the metropolitan areas of Lisboa and Porto [50].

Table 3 details some demographic characteristics of the mainland, evidencing that almost half (about 47%) of the mainland population is concentrated in less than 4% of the country area, previously confirmed by Fernandes and Seixas [51] and Guimarães and co-workers [52]. Additionally, a continued decrease of population density, a recurrent tendency being observed for decades and mentioned by Guimarães and co-workers [52], and Knoema Global Database [53], is more accentuated in predominantly rural regions. Another relevant phenomenon that may also be observed from Table 3 is the progressive ageing of the country population, confirmed by Rodrigues and co-workers [54], which is rather pertinent in predominantly rural regions, currently with a ratio of 306.7 elderly per 100 young people. 

All these demographic imbalances are extremely significant due to the direct impact on distribution (localization) of healthcare services, which tend to be concentrated in higher-populated regions, as stated by Santana [55]. In contemporary societies, healthcare services distribution should be in accordance with communities’ needs. However, at present, the Portuguese NHS is aligned with the “Inverse Care Law”, primarily defined by Tudor Hart [56,57] that results in a mismatch between the lack of specialized health care services and populations’ healthcare needs, particularly in isolated rural regions as Santana refers [55]. This demographic profile stands as a challenge that has not yet been taken into account for the definition of public health policies, as has been stated by Vidal and co-workers [30,58]. 

**Figure 2:**
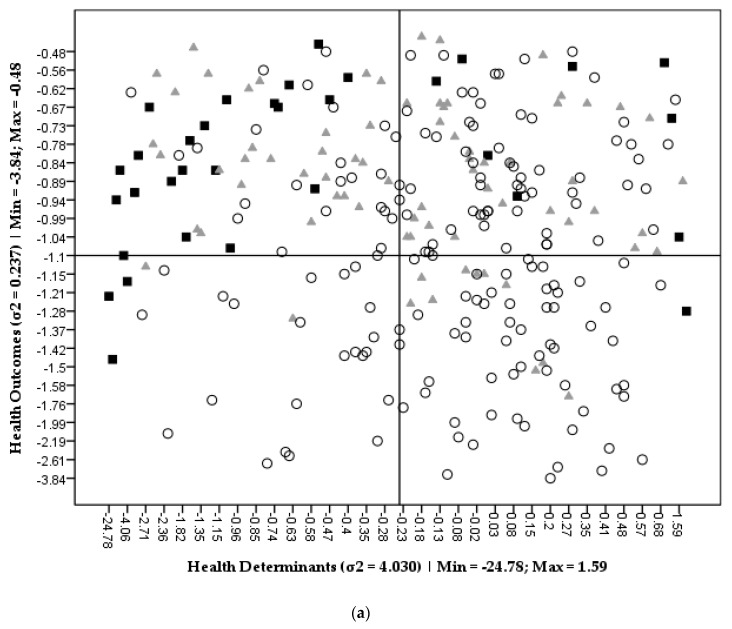

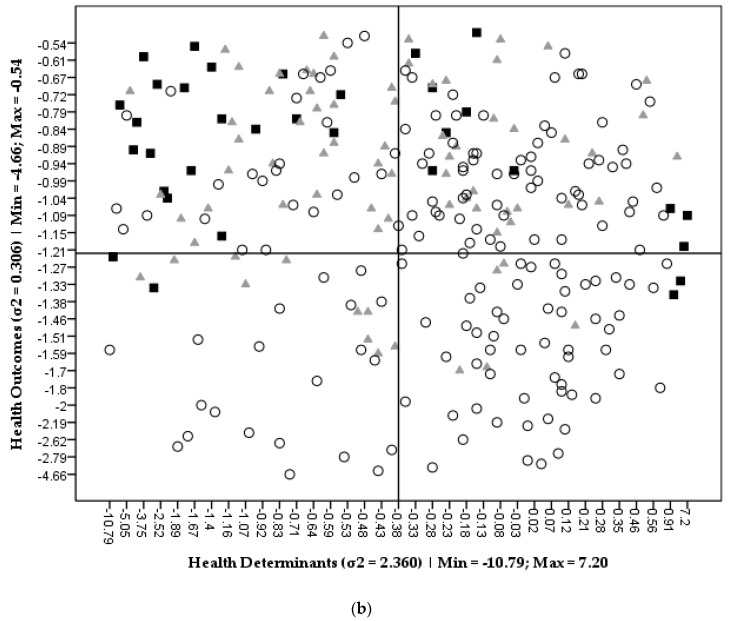

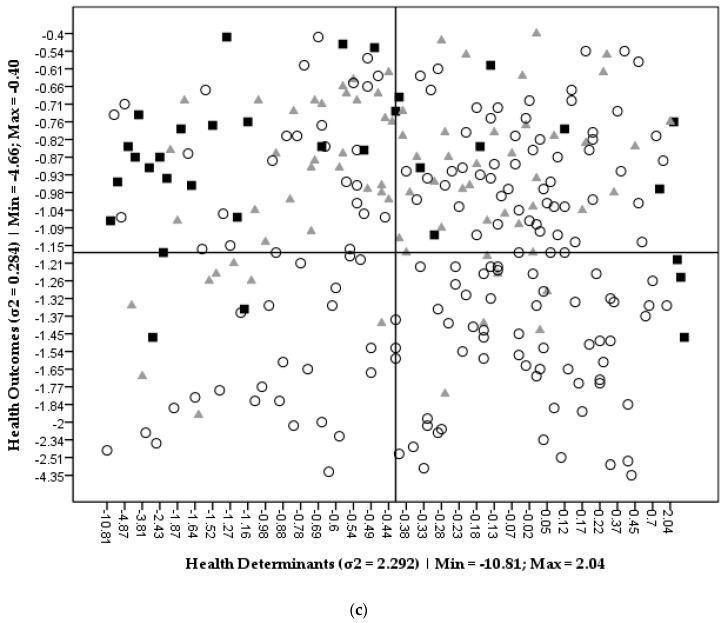
Scatter plot of health determinants (x-axis) and health outcomes (y-axis) value-scores by territory typology and corresponding variance (σ^2^) and minimum/maximum scores: (**a**) 2009; (**b**) 2015; (**c**) 2017/2018. Each symbol represents one municipality. ■ Predominantly urban; ▲ semi-urban; ○ predominantly rural.

Figure 2 graphically represents *SEHVI* scores for all mainland municipalities (N = 278) as functions of their correspondent health determinants and health outcomes. Each year of analysis (2009, 2015, 2017/2018) has an individual diagram where municipalities are represented by different symbols according to the population density definition adopted. Additionally, Table 4 summarizes the means and their correspondent standard deviation values of the *SEHVI* scores for the aggregated municipalities typologies (PU, SU, PR). Table 4 also includes results from the application of the previously referred statistical tests (ANOVA with Tukey H.S.D.).

Each diagram of Figure 2 is partitioned in four quadrants resulting from the axis division that was automatically defined by IBM^®^ SPSS^®^ Statistics vs. 25.0 software using “Jenks Natural Breaks” data analysis tool. Therefore, the scale of the *y-* and *x*-axis intervals are different for each year of analysis; thus, minimum and maximum of each interval are referred in the legends. It is worth to recall that, for each year of analysis, *SEVHI* has a reference value of zero corresponding to the national average. For every municipality, the analysis of the index score evolution in time must maintain this perspective of the national reference of zero, so absolute comparisons are not possible with this methodology as the reference point (national average) changes from one year to another. This is the reason that axis intervals change from one year to the other. However, monitoring in time is always possible comparatively to the national reference as, for each year of analysis, the upper right quadrant of each diagram includes the municipalities with the best performance both in health determinants and in health outcomes, while the lower left quadrant contains the municipalities with the worst performance in the same parameters. This analysis is equal for any year of the study and the diagrams allow a better of reading of the statistical results presented in Table 4. 

Higher scores variance identified in health determinants were more intense in 2009 (σ^2^ = 4.030). A variance decrease in health determinants from 2009 to present is also noticeable, evidencing less disparities between territory typologies (F = 1.80; *p* = 0.17). In the lowest position, at the lower left quadrant, are mainly rural (PR) municipalities with lower scores. This could be explained by the lack of healthcare services in those regions, aggravated by their communities demographic characteristics: Aged groups, less educated, and with lower income, as mentioned by previous studies [31,59,60]. On the other hand, the upper left quadrant includes most of the urban (PU) municipalities with the higher health outcomes scores (corresponding to less mortality) though with lower health determinants scores. These municipalities are those who have specialized healthcare services and human resources and, accordingly, have better health outcomes, confirmed by Mackenbach and co-workers [10]. However, pollution, urban waste production, and energy consumption puts them at the bottom of health determinants scores [61,62,63]. In the lower right quadrant are, almost entirely, rural municipalities with lower health outcomes scores but with higher health determinants scores. These municipalities, although having poor access to better health care services and education and work opportunities, are less exposed to harmful environmental conditions, experiencing better air quality, less road traffic, and population pressure, which are in accordance with past studies [63,64]. Finally, most of the semi-urban (SU) municipalities are positioned in both upper quadrants of all diagrams, thus showing better health outcomes, independently of health determinants scores. It seems that these types of communities (SU) are able to manage the “best of both worlds”: On one hand, usually they are not very distant from urban centers, and so may profit from access to specialized health care and education services, and work opportunities; on the other side, they are not at the center of pollution and intensive traffic, more frequently found in urban areas, leaving these populations exposed to a better environmental quality [64]. According to Table 4, health outcomes scores are better in predominantly urban (PU) and semi-urban (SU) areas for all years of analysis, namely: 2009 (F = 18.7; *p* < 0.01), 2015 (F = 20.3; *p* < 0.01), and 2017/2018 (F = 17.5; *p* < 0.01). Health determinants scores are only significantly different in 2009 (higher in rural (PR) areas; F = 12.7; *p* < 0.01). As can be seen in Table 4, there were no statistically significantly differences in *SEHVI* scores among territory typologies in the three time periods (*p* > 0.05).

**Figure 3:**
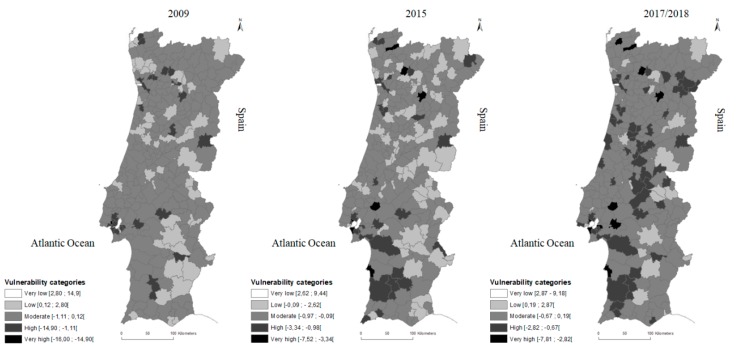
SEHVI cartographic representation for three years under analysis.

A cartographic representation of all *SEHVI* scores was chosen due to the large number of municipalities (N = 278) and is presented in Figure 3. Accordingly, in Table 5, for each year of analysis, municipalities are grouped in vulnerability categories defined by *SEHVI* scores according to scales presented in Figure 3. Additionally, Table 5 presents the distribution of municipalities according to population density (PU, SU, PR) and their correspondent share of mainland population in each vulnerability category.

It is worth to remark that from 2009 to the present, more than half of the mainland population experienced “Moderate” vulnerability to health (53.6% in 2009, 51.2% in 2015, and 55.0% in 2017/2018). According to Santana and co-workers [65,66] these results are aligned with the Portuguese health gains and health promotion translated by recent public health policies.

From 2009 to 2015 (maintained in 2017/2018), the share of population with “Very low” health vulnerability increased. Nevertheless, this category includes only the three country main cities. On the other hand, in the same period of analysis, the share of population exposed to “very high” vulnerability has increased from only one municipality in 2009 (corresponding to the municipality with the highest population density) to a total of nine municipalities and to 4.8% share of population. However, most of the rural municipalities (PR) experience “Moderate” and “High” health vulnerability. Only urban municipalities experience “Very low” health vulnerability, scoring the highest *SEHVI* values, which is in accordance with the study developed by Simões and co-workers [67]. Semi-urban municipalities (SU) scores remain mostly in the “Moderate” vulnerability category, but the number of SU municipalities in the “Low” vulnerability is decreasing while the number in “High” and “Very high” vulnerability categories are increasing.

The spatial distribution of *SEHVI* index scores are cartographically represented in Figure 3. for the three analysed years. An increase of regions in dark greyscale and black from 2009 to present clearly indicates a worsening in health vulnerability, which is in agreement with results of Table 5. The highest *SEHVI* scores, represented in white, remain scarce in the period of analysis corresponding only to three of the country main cities. These cities have the most important academic institutions and benefit from the most complete and specialized healthcare infrastructures of the country, which are very important determinants to life conditions. In addition, these cities count on technological development and innovation, that contribute to better qualified work positions [68].

## 4. Conclusions

The major contribution of this study is the development of an index to evaluate health vulnerability as a function of health determinants and health outcomes at a local scale. Variables composing the index are aligned with the SDG 3—“Health promotion”—and SDG 10—“Inequalities”—but are limited by data availability from national databases. The 2030 UN Agenda principle “leaving no one behind” calls for universal commitment so there was a need to provide for an instrument to monitor progress in sustainable development at the communities’ level. *SEHVI* concerns of populations’ health and life conditions to promote “Health and wellbeing to all at all ages”. Taking into account communities’ specificities, the index provides information to map and identify communities’ vulnerability and health determinants gaps that need to be overcome in order to assist development of sustainable public health policies that are oriented to people’s needs.

Although Portugal is a small, predominantly rural country, differences were found between urban and rural municipalities, suggesting inequalities and higher vulnerability in these latest communities. The mainland considerable diversity becomes clear through the analysis of health determinants and outcomes and with *SEVHI* results. During the period of 2009 to the present, predominantly urban municipalities always performed better (“very low” and “low” vulnerability) in health outcomes than predominantly rural communities where mortality is higher. This persistent pattern observed in this study translates a real inequality in life conditions of more vulnerable and aged rural communities. This is a matter of concern that should call for specific public health policies at a local level, specifically oriented to isolated rural municipalities. 

This work also aims to contribute to stress the importance of adopting tools to monitor the effectiveness of public health policies at the local level, because promoting wellbeing and good life conditions should be a shared responsibility of municipalities’ leaders and local communities’ stakeholders, besides central government.

This study provides a validated tool to quantify a very complex subject, which is the population’s health vulnerability. The main strength of *SEHVI* relates to its concept: The simplicity of a single score outcome being tailored designed to focus on local communities and territories, allowing a local approach. *SEHVI* is based on credible data sources (National Statistics Institute and other public departments); is easily adaptable to other geographic scales; allows the follow-up of local interventions; and puts in evidence the urgent need to improve the quality of data collected and its management. Besides these strengths, *SEHVI* has some limitations, that are, however, extrinsic to its concept and design: The index is restrained by the scarcity of data at local level that left the Portuguese archipelagos of Açores and Madeira out of this study. Lack of specific data made it impossible to cover the progress on all UN sustainable development goals; in the same line, the selection of variables is dependent on data availability, both in time and in type. The absence of data on morbidity of noncommunicable diseases, especially the most common such as diabetes, obesity, or high blood pressure, is an important limitation of the study; however, as previously referred, this kind of information disaggregated at the local level are not available in official databases. The relevance of composite indicators to measure (in objective and quantifiable manners) progress in several dimensions of life has been internationally recognized by several researchers and institutions because indicators enable the quantification of multidimensional concepts that cannot be translated in just one indicator. Also, indexes are helpful tools to identify trends, to public communication, and assist policy decisions.

## Figures and Tables

**Table 1:** Variables selected to compose socioeconomic health vulnerability index *(SEHVI)*.

Dimension		Indicators	Time Series Available	Polarity
Health Outcomes	Mortality	1 Infant mortality rate (‰)	2009–2017	-
		2 Deaths by HIV and Tuberculosis (No.)	2009–2017	-
		3 Deaths by circulatory Diseases (No.)	2009–2017	-
		4 Deaths by Tumors (No.)	2009–2017	-
		5 Deaths by Diabetes (No.)	2009–2017	-
		6 Deaths by Respiratory Diseases (No.)	2009–2017	-
		7 Suicide (No.)	2009–2017	-
Health Determinants	Healthcare Resources	8 Number of health care professionals (No.)	2009–2017	+
		9 Number of hospitals (No.)	2009–2016	+
		10 Number of primary health care centers (No.)	2009–2012	+
	Education	11 Number of illiterate persons (No.)	2001;2011	-
		12 Number of persons enrolled in basic education (No.)	2009–2017	+
		13 Number of persons enrolled in pre-graduate studies (No.)	2009–2017	+
		14 Number of persons in higher education (No.)	2009–2017	+
		15 Number of persons in lifelong learning (No.)	2008–2017	+
	Water and Sanitation	16 Population connected to public water supply systems (%)	2009	+
		17 Population connected to sewerage systems (%)	2009–2016	+
		18 Water supplied/consumed (m^3^)	2009–2016	-
		19 Water quality for human consumption (%)	2009–2016	+
	Air Pollution and Land	20 NO_x_ emissions (ton/km^2^)	2009;2015	-
		21 PM_10_ emissions and PM_2.5_ emissions (ton/km^2^)	2009;2015	-
		22 CO_2_ emissions (ton/km^2^)	2009;2015	-
		23 Burnt Area (ha)	2009–2017	-
	Waste	24 Selective urban waste collection (ton)	2009–2016	+
		25 Undifferentiated urban waste collection (ton)	2009–2016	-
		26 Incinerators and Landfills (No.)	2014;2016	-
	Safety	27 Deaths by car accidents (No.)	2009–2017	-
		28 Crimes registered (No.)	2009–2017	-
	Housing	29 Non-conventional dwellings (No.)	2001;2011	-
		30 Buildings constructed before 1960 (No.)	2001;2011	-
	Employment and Income	31 Inactive young population (15–34 years) (No.)	2001;2011	-
		32 Average monthly salary (Euro)	2009–2016	+
		33 Unemployed (No.)	2009–2018	-
	Social Protection	34 Social Security beneficiaries of Guaranteed Minimum Income and Social Integration Benefit (No.)	2009–2018	-
	Cultural and social participation	35 Expenditures on cultural and creative activities of municipalities (Euro)	2009–2017	+

**Table 2:** Factor analysis for *SEHVI*.

Components	Individual Indicators	I	II	III	IV	V	VI	VII	VIII	IX	*C*	(α) *
**12.9%**	30 Buildings constructed before 1960	0.83									0.87	0.79
	2 Deaths by HIV and Tuberculosis	0.31									0.41	0.81
	8 Number of health care professionals	0.70									0.80	0.81
	9 Number of hospitals	0.90									0.91	0.79
	14 Number of persons in higher education	0.71									0.82	0.81
	35 Expenditures on cultural and creative activities of municipalities	0.86									0.87	0.78
**11.4%**	22 CO_2_ emissions		0.93								0.92	0.77
	20 NO_x_ emissions		0.93								0.96	0.76
	21 PM_10_ emissions and PM_2.5_ emissions		0.91								0.90	0.77
	10 Number of primary health care centers		0.71								0.95	0.79
	11 Number of illiterate persons		0.65								0.89	0.79
**9.7%**	26 Incinerators and Landfills			0.46							0.44	0.81
	3 Deaths by circulatory Diseases			−0.80							0.69	0.81
	6 Deaths by Respiratory Diseases			−0.69							0.56	0.81
	4 Deaths by Tumors			−0.81							0.68	0.81
	12 Number of persons enrolled in basic education			0.66							0.76	0.81
	5 Deaths by Diabetes			0.63							0.62	0.81
	33 Unemployed			0.56							0.62	0.81
**8.5%**	18 Water supplied/consumed				0.65						0.50	0.81
	24 Selective urban waste collection				0.84						0.74	0.81
	25 Undifferentiated urban waste collection				0.85						0.76	0.81
	28 Crimes registered				0.65						0.67	0.81
	31 Inactive young population (15–34 years)				−0.41						0.54	0.81
**6.8%**	13 Number of persons enrolled in pre-graduate studies					0.77					0.73	0.81
	15 Number of persons in lifelong learning					0.81					0.69	0.81
**5.9%**	23 Burnt Area						0.61				0.50	0.81
	16 Population connected public water supply systems						−0.58				0.51	0.81
	34 Social Security beneficiaries of Guaranteed Minimum Income and Social Integration Benefit						0.68				0.75	0.81
	32 Average monthly salary						−0.38				0.58	0.81
**4.6%**	29 Non-conventional dwellings							0.66			0.61	0.81
	17 Population connected to sewerage systems							0.47			0.46	0.81
**4.3%**	7 Suicide								0.79		0.70	0.81
	27 Deaths by car accidents								0.63		0.48	0.81
**3.5%**	19 Water quality for human consumption									0.50	0.40	0.81
	1 Infant mortality rate									0.77	0.66	0.81

Notes to the Table: Extraction method - Principal components. Varimax rotation with Keiser normalization. Extraction criterion: Eigenvalues > 1. Total variance explained by extracted components: 68.2%; KMO = 0.77; Bartlett’s test: χ^2^ = 7064.2, *p* < 0.001; *C* - Communalities; * Cronbach’s alpha (α) if item is removed; Global Cronbach’s alpha: α = 0.81.

**Table 3:** Demographic characteristics of the three territory typologies.

Municipality Typologies (N)	Area (km^2^)	Population (Inhabitants)			Population Density (Inhabitants/km^2^)			Ageing Index		
		2009	2015	2017/2018	2009	2015	2017/2018	2009	2015	2017/2018
PU (33)	3478 (3.9%)	4,676,005 (46.5%)	4,627,128 (47.0%)	4,630,237 (47.2%)	1344.5	1330.4	1331.3	113.3	130.6	138.3
SU (76)	15,641 (17.6%)	3,162,391 (31.4%)	3,130,700 (31.8%)	3,115,298 (31.8%)	202.2	200.2	199.2	137.3	163.1	177
PR (169)	69,982 (78.5%)	2,217,205 (22.0%)	2,096,697 (21.3%)	2,055,636 (21.0%)	31.7	30.0	29.4	250	288	306.7

Notes to the table: PU—predominantly urban; SU—semi-urban; PR—predominantly rural.

**Table 4:** Descriptive statistics by territory typologies.

Time	Territory Typology	Outcomes		Determinants		SEHVI	
		M ± SD	M ≠	M ± SD	M ≠	M ± SD	M ≠
2009	PU	−0.83 ± 0.25	M_PU_₋M_SU_ = 0.05	−1.97 ± 5.33	M_PU_₋M_SU_ = −1.63 *	−0.62 ± 4.00	M_PU_₋M_SU_ = −0.43
	SU	−0.88 ± 0.25	M_PU_₋M_PR_ = 0.38 *	−0.34 ± 0.81	M_PU_₋M_PR_ = −1.85 *	−0.19 ± 0.63	M_PU_₋M_PR_ = −0.41
	PR	−1.21 ± 0.55	M_SU_₋M_PR_ = 0.33 *	−0.13 ± 0.60	M_SU_₋M_PR_ = −0.22	−0.21 ± 0.47	M_SU_₋M_PR_ = 0.02
		*F* = 18.7 *		*F* = 12.7 *		*F* = 1.21	
2015	PU	−0.89 ± 0.23	M_PU_₋M_SU_ = 0.08	−0.99 ± 3.15	M_PU_₋M_SU_ = −0.43	0.06 ± 3.15	M_PU_₋M_SU_ = 0.43
	SU	−0.97 ± 0.29	M_PU_₋M_PR_ = 0.46 *	−0.56 ± 0.89	M_PU_₋M_PR_ = −0.61	−0.37 ± 0.69	M_PU_₋M_PR_ = 0.50
	PR	−1.35 ± 0.63	M_SU_₋M_PR_= 0.38	−0.39 ± 1.25	M_SU_₋M_PR_ = −0.17	−0.44 ± 0.89	M_SU_₋M_PR_ = 0.07
		*F* = 20.3 *		*F* = 2.23		*F* = 1.94	
2017/2018	PU	−0.91 ± 0.25	M_PU_₋M_SU_ = 0.05	−0.97 ± 3.07	M_PU_₋M_SU_ = 0.43	0.07 ± 3.06	M_PU_₋M_SU_ = 0.41
	SU	−0.96 ± 0.28	M_PU_₋M_PR_ = 0.40 *	−0.52 ± 0.85	M_PU_₋M_PR_ = 0.50	−0.34 ± 0.68	M_PU_₋M_PR_ = 0.52
	PR	−1.31 ± 0.61	M_SU_₋M_PR_ = 0.35 *	−0.42 ± 1.27	M_SU_₋M_PR_ = 0.07	−0.45 ± 0.91	M_SU_₋M_PR_ = 0.11
		*F* = 17.5 *		*F* = 1.95		*F* = 1.17	

Notes to the table: PU—predominantly urban (N = 33); SU—semi-urban (N = 76); PR—predominantly rural (N = 76); M—mean; SD—standard deviation; M ≠—mean difference; * significant at 0.01 level; *F*—One-way ANOVA.

**Table 5:** Distribution of mainland municipalities number, according to population density (predominantly urban (PU), semi-urban (SU), predominantly rural (PR)) and their corresponding share of population for each year of analysis. Results are grouped by vulnerability categories defined as a function of *SEHVI* scores (please refer to Figure 3 legend).

SEHVI		2009					2015					2017/2018				
		Municipalities (N)					Municipalities (N)					Municipalities (N)				
		PU	SU	PR	Total	Population (%)	PU	SU	PR	Total	Population (%)	PU	SU	PR	Total	Population (%)
**Vulnerability categories**	Very low	1	0	0	1	2.4	3	0	0	3	7.6	3	0	0	3	7.6
	Low	9	16	26	51	21.7	8	17	46	71	21.2	5	5	13	23	9.9
	Moderate	12	54	135	201	53.6	11	54	105	170	51.2	10	60	118	188	55.0
	High	10	6	8	24	20.6	9	4	14	27	16.0	13	9	33	55	22.7
	Very high	1	0	0	1	1.7	2	1	4	7	4.1	2	2	5	9	4.8
	Total	33	76	169	278	100	33	76	169	278	100	33	76	169	278	100

Notes to the table: PU—predominantly urban; SU—semi-urban; PR—predominantly urban.
